# Long-term low-dose ethanol intake improves healthspan and resists high-fat diet-induced obesity in mice

**DOI:** 10.18632/aging.103401

**Published:** 2020-07-08

**Authors:** Yan Diao, Junhui Nie, Peizhu Tan, Yuchen Zhao, Tingting Zhao, Jiajie Tu, Heng Ji, Yuwei Cao, Zhaojing Wu, Huan Liang, Hui Huang, Yanze Li, Xu Gao, Lingyun Zhou

**Affiliations:** 1 Department of Biochemistry and Molecular Biology, Harbin Medical University, Harbin, China; 2 Department of Clinical Laboratory, Heilongjiang Province Hospital, Harbin, China; 3 Translational Medicine Center of Northern China, Harbin, China; 4 Department of Clinical Laboratory, Harbin Medical University Cancer Hospital, Harbin, China; 5 Key Laboratory of Preservation of Human Genetic Resources and Disease Control in China, Ministry of Education, China

**Keywords:** ethanol, alcohol, thermogenic activity, obesity, insulin resistance

## Abstract

Numerous epidemiological studies have reported that moderate alcohol drinking has beneficial effects. However, few studies have focused on the beneficial effects of ethanol, the common component in alcoholic beverages. Here we fed the C57BL/6 mice with 3.5% v/v ethanol as drinking water substitute to investigate the effects of long-term low-dose ethanol intake *in vivo*. We evaluated the metabolic rate and mitochondrial function of the long-term low-dose ethanol-intake (LLE) mice, assessed the exercise ability of LLE mice, and fed the LLE mice with a high-fat diet to investigate the potential impact of ethanol on it. The LLE mice showed improved thermogenic activity, physical performance, and mitochondrial function, as well as resistance against the high-fat diet-induced obesity with elevated insulin sensitivity and subdued inflammation. Our results suggest that long-term low-dose ethanol intake can improve healthspan and resist high-fat diet-induced obesity in mice. It may provide new insight into understanding the protective effects of moderate alcohol drinking.

## INTRODUCTION

Alcoholic beverages among the most common worldwide beverages in people’s daily life. As the common component of beer, wine and liquor, alcohol or ethanol, is controversial on the risk-benefit balance of consumption. In many studies, excessive consumption of alcohol has been proved to be associated with increased risks in more than 60 types of diseases, such as cardiovascular disease, alcoholic liver disease, and cancer [1, 2]. As a result, the harmful effects of drinking are commonly concerned, whereas the benefits of moderate alcohol consumption are less appreciated. Recently, some epidemiological studies showed that moderate alcohol intake could lower all-cause mortality compared to non-drinkers and heavy drinkers [3–5]. Further epidemiological reports suggested that moderate daily alcohol intake might reduce the risk of cognitive decline in women and promote the release of brain acetylcholine [6, 7]. Multiple studies have also shown that moderate drinking has cardiovascular protective effects and is linked to reduced risks of heart failure [8, 9]. Regrettably, concerned predominantly with the risks of excessive alcohol consumption, scientists have paid little attention to the beneficial effects of moderate alcohol intake. Determining the benefits of moderate alcohol intake is therefore important for people to drink safely and healthily without having alcohol-related issues.

Obesity is a leading cause of morbidity and mortality worldwide. Overweight and obesity trigger metabolic disorders that are accompanied by manifestations such as dyslipidemia, insulin resistance, and chronic systemic inflammation [10]. Obesity is also associated with increased risks of cardiovascular disease and other age-related diseases [11]. All of the abovementioned diseases caused by obesity bear adverse impacts on health and generally reduce lifespan. Intriguingly, epidemiologic studies showed the fact that moderate alcohol intake is linked to lower risks of cardiovascular disease and metabolic-related disease [4, 9, 12, 13]. This raises the possibility of a beneficial role for moderate alcohol intake in other age-related diseases. Some previously published studies have suggested that moderate consumption of 30 g/d of alcohol (2 drinks per day) is beneficial to insulin sensitivity and triglyceride concentration in nondiabetic postmenopausal women and could decrease the risk of diabetes and improve insulin resistance [14, 15]. In another study, Wang et al. observed an inverse relationship between moderate alcohol consumption and the risk of becoming overweight or obese in a cohort of middle-aged and older women during 12.9 years of follow-up [16]. However, there is still no definitive answer to whether ethanol plays a key role in these beneficial processes.

Despite the progress in the epidemiological research, there have been few studies showing the effectiveness of moderate alcohol intake on the improve healthspan in mammals, leading us to study chronic low-dose ethanol supplementation in laboratory mice. Our previous study has shown that chronic moderate alcohol intake accelerates SR-B1 mediated reverse cholesterol transport [17]. We subsequently hypothesized that moderate alcohol intake might improve the physical condition of mice. Cohorts of male C57BL/6 mice were provided with a standard diet or an otherwise equivalent high-fat diet (HFD, 45% of caloric fat), each diet supplemented with 3.5% ethanol from week 8 to the end of life. The physical and physiological performances of long-term low-dose ethanol intake (LLE) mice were evaluated in this research.

## RESULTS

### Long-term low-dose ethanol intake increases thermogenic activity and improves physical performance of mice

**Figure 1:**
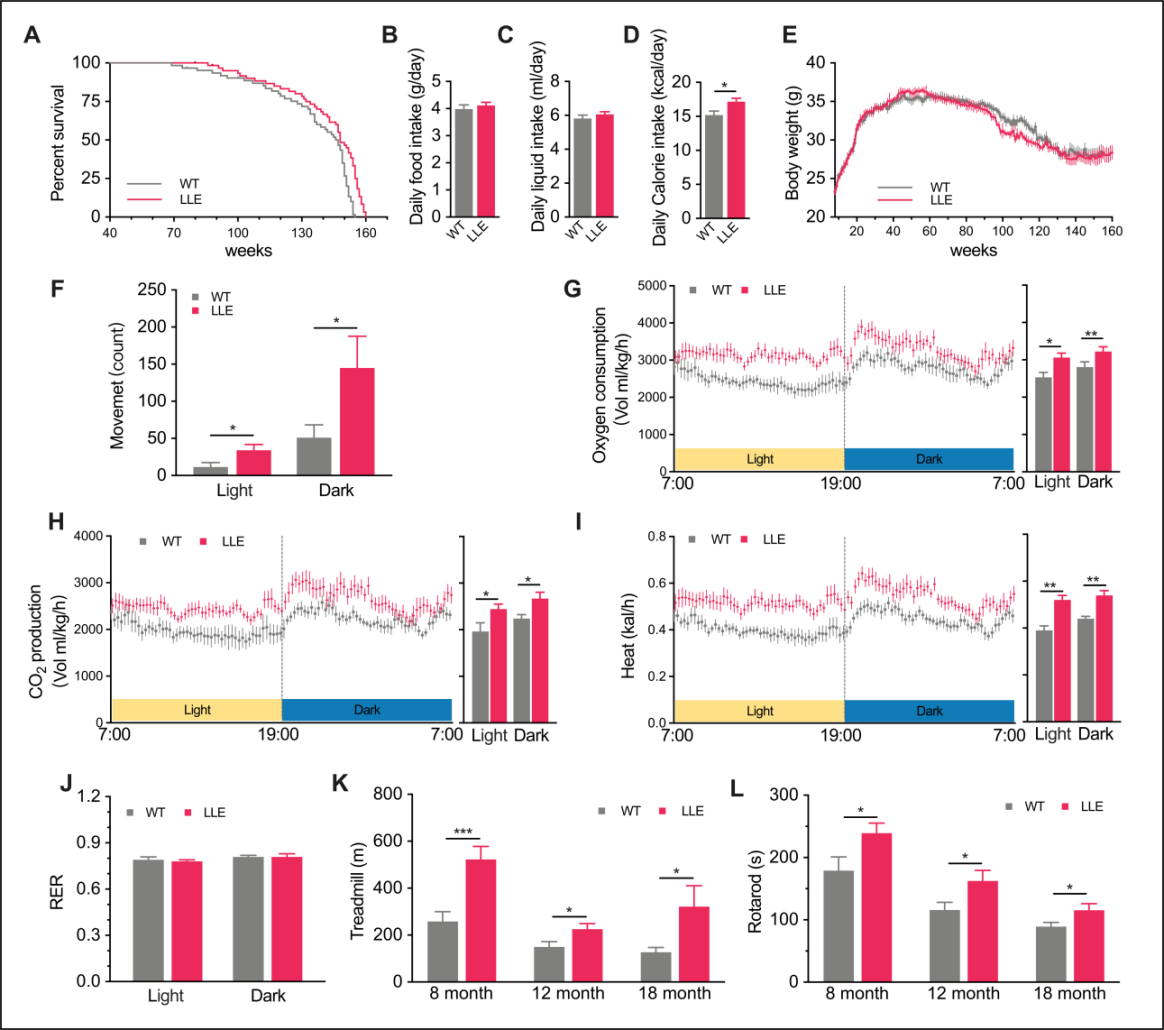
**Increased thermogenic activity and improved physical performance in LLE mice.** (**A**) Kaplan-Meier survival curve for long-term low-dose (3.5% v/v) ethanol intake (LLE) mice and untreated wild-type (WT) mice. (n=60 for each group). (**B**–**D**) Daily food intake, daily liquid intake, and daily calorie intake of 32-week-old LLE and WT mice (n=25 for each group). (**E**) Change in body weight (n= for each group). (**F**) X-axis movement of 44-week-old LLE and WT mice, an indicator of spontaneous locomotor activity in CLAMS system (n=8 for each group). (**G**–**J**) Oxygen consumption rate, CO_2_ production rate, energy expenditure rate, and respiratory exchange ratio (RER) of 44-week-old LLE and WT mice (n=8 for each group). (**K**–**L**) Distance run on treadmill and time to fall from an accelerating rotarod measured at 8, 12, 18 months, (n=10 for each group) Data are presented as mean ± SEM. *, *P*<0.05, **, *P*< 0.01, ***, *P*<0.001.

To determine the long-term effects of moderate ethanol intake in mice, the drinking water was supplemented with 3.5% (v/v) ethanol from 8 weeks after birth until the end of life. The survival curve of the LLE mice was slightly shifted to the right (Figure 1A), leading to a 4.42% extension of mean lifespan (Chi square=5.896, *P*=0.0152, in Gehan–Breslow survival test). Thorough post mortem anatomical and histopathological studies did not show noticeable differences between the LLE mice and the WT mice in their pathology or cause of death. Neither were their plasma ALB, TP, ALT, AST, or BUN levels significantly different, indicating comparable organ functions (Table 1). In comparison, our previous studies showed that a high-level ethanol intake (15% v/v) could cause the elevation of ALT and AST, and induce alcoholic fatty liver disease [18–20]. While cognition and learning which can be impaired by a large amount of alcohol intake, water maze test indicated that the cognitive ability of the LLE mice was slightly improved (Supplementary Figure 1A–1D). The LLE mice did not show the cognitive impairment that alcoholism might have caused, and instead exhibited signs of improved cognition that was reported previously [6].

**Table 1:** Serum parameters after 12 weeks experimental diets.

	**WT**	**LLE**	**HFD**	**LLE+HFD**
LDH (U/L)	559.5±105.03	473±45.25	686±5.29	516±71.18
AST (U/L)	87.00±25.26	85±43.84	86±10.69	85±12.36
ALT (U/L)	44.83±15.16	37.86±6.91	62±13.11^#^	30±5.7*
ALB (U/L)	32±4.52	31.67±1.97	28.67±0.58	29.6±1.34
TP (g/L)	50.87±3.67	50.47±1.53	49.07±0.96	49.64±2.0
GLU (mmol/L)	8.39±1.09	7.9±1.16	13.07±1.60^##^	9.3294±1.40**
BUN (mmol/L)	8.365±0.41	8.19±0.28	7.0633±0.6^##^	7.304±0.70
TG (mmol/L)	0.97±0.39	0.815±0.01	1.374±0.31^#^	0.85±0.49**
T-CHO (mmol/L)	2.51±1.09	2.7±0.15	4.1183±0.36^##^	3.32±0.53*
HDL-C(mmol/L)	2.192±0.4	2.31±0.48	4.104±0.18^##^	3.22±0.54**
LDL-C(mmol/L)	0.20±0.02	0.24±0.01	0.396±0.09^##^	0.258±0.04**

Value are presented as mean ± SEM. WT, n=12; LLE, n=17; HFD, n=18; LLE+ HFD, n=17. # *P*<0.05 vs WT, ## *P*<0.01 vs WT, * *P*<0.05 vs HFD, ** *P*<0.01 vs HFD. LDH, lactate dehydrogenase; AST, aspartate aminotransferase; ALT, alanine aminotransferase; ALB, albumin; TP, total protein; GLU, glucose; CRE, creatinine; BUN, blood urea nitrogen; TG, triglycerides; T-CHO, total cholesterol; HDL-C, high density lipoprotein cholesterol; LDL-C, low density lipoprotein cholesterol.

To determine if low-dose ethanol intake influence the dietary habit, the daily food and liquid intake was recorded. There were no noticeable differences between the two groups in the dietary habit (Figure 1B, 1C), but the calorie intake of LLE mice was slightly higher than WT mice (Figure 1D) because the ethanol can be a source of energy. Intriguingly, the higher calorie intake did not lead to weight gain in LLE mice (Figure 1E). However, it led us to wonder if the daily activity of mice was altered. Comprehensive Lab Animal Monitoring System was subsequently used to monitor mice activity and metabolic rates. With the supplement of ethanol, the LLE mice tend to be more active (Figure 1F). The higher oxygen consumption (Figure 1G), carbon dioxide production (Figure 1H) and heat production (Figure 1I) in both light phase and dark phase indicated that the basal metabolic rates of the LLE mice increased. Also noteworthily, the respiratory exchange ratio (RER) of two groups are almost the same (Figure 1J), suggesting comparable rates of oxidation of fat and carbohydrates in this state.

Given the appreciable effects of long-term low-dose ethanol intake on the metabolic rate, it was necessary to determine whether the physical performance, which reflects the state of health, was preserved in the LLE mice. One way to assess this was to measure endurance and motor coordination, which we examined by using a rotarod and a treadmill. Interestingly, our observations showed that the LLE mice performed significantly better than WT mice (Figure 1K, 1L). These results indicate that the long-term low-dose ethanol intake will enhance the physical performance of the mice rather than impair it.

### Long-term low-dose ethanol intake enhances the mitochondrial function of mice

**Figure 2:**
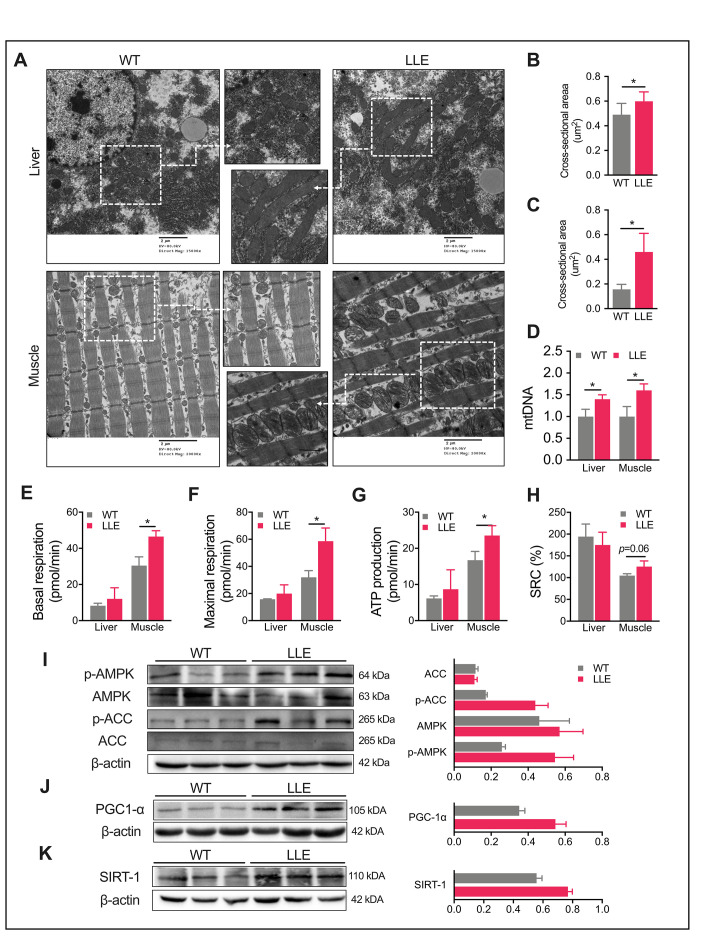
**Enhanced mitochondrial function in LLE mice.** (**A**) Electron micrographs of liver and gastrocnemius muscle of 32-week-old mice (Scale bar = 2μm). (**B**–**C**) Mitochondrial average cross-sectional area of liver and gastrocnemius muscle of 32-week-old mice (n=3 for each group). (**D**) Quantification of mitochondrial DNA (mtDNA) of 32-week-old mice by real-time PCR. (n=3 for each group). (**E**–**H**) Basal respiration rate, maximal respiration rate, ATP production, and spare respiration capacity (SRC) of liver and gastrocnemius muscle of 32-week-old mice (n=3 for each group). (**I**) Total and phosphorylated levels of AMPK and its downstream target, acetyl-CoA carboxylase (ACC) in LLE mice liver. (**J**–**K**) Western blot analysis and relative quantification of hepatic PGC1a and hepatic SIRT1. Data are presented as mean ± SEM. *, *P*< 0.05, **, *P*< 0.01, ***, *P*< 0.001.

Increased daily activity and improved physical performance of the LLE mice implied enhanced mitochondrial function. We next examined the mitochondria in liver and muscle using electron microscopy. A markedly increased number of mitochondria in the liver and gastrocnemius muscle of LLE mice was observed in the images, displaying visibly larger mitochondrial structures with an increased presence of cristae in the LLE mice (Figure 2A–2C). Mitochondrial DNA (mtDNA) content was also quantified by PCR. As expected, mtDNA markedly increased in the LLE mice (Figure 2D). To further evaluate the mitochondrial function of the LLE mice, the oxygen consumption rate (OCR) of the liver and the muscles were examined. The basal oxygen consumption rate was elevated in the muscles of the LLE mice (Figure 2E). Following the addition of oligomycin and FCCP, the muscles of LLE mice showed an increased ATP production and maximal respiration (Figure 2F, 2G). Spare respiratory capacity (SRC), the extra mitochondrial capacity available in cell to produce energy under conditions of increased work or stress, was also slightly increased (*P*=0.06, Figure 2H). Taken together, these results indicate that mitochondrial function was enhanced under the long-term ethanol treatment in the LLE mice.

AMPK has emerging roles in the regulation of both mitochondrial metabolism and dynamics [21, 22]. It participates in a variety of signaling and transcriptional pathways that mediate the energy metabolism and induces mitochondrial biogenesis, one of which being the promotion of mitochondrial biogenesis of SIRT1 and gene expression via PGC1a in an AMPK-dependent manner. We next explored whether AMPK activity was influenced by ethanol in the liver. The results showed that the LLE mice had a strong tendency toward the induced phosphorylation of AMPK (Figure 2I), which led to the increased phosphorylation of acetyl-CoA carboxylase (ACC) at Ser-79, the downstream target of AMPK (Figure 2I). Mitochondrial biogenesis in liver and muscle is mainly regulated by the transcriptional coactivator PGC1a, the activity of which is positively regulated by SIRT1-mediated deacetylation [23, 24]. Therefore, we tested the expression of SIRT1 and PGC1a and found increased expressions of both proteins in the liver of the LLE mice when compared with WT mice (Figure 2J, 2K). These data suggested that moderate ethanol intake could improve mitochondrial function such that the LLE mice showed enhanced endurance with respect to running and other health benefits.

### Distinct gene expression pattern of the LLE mice

**Figure 3:**
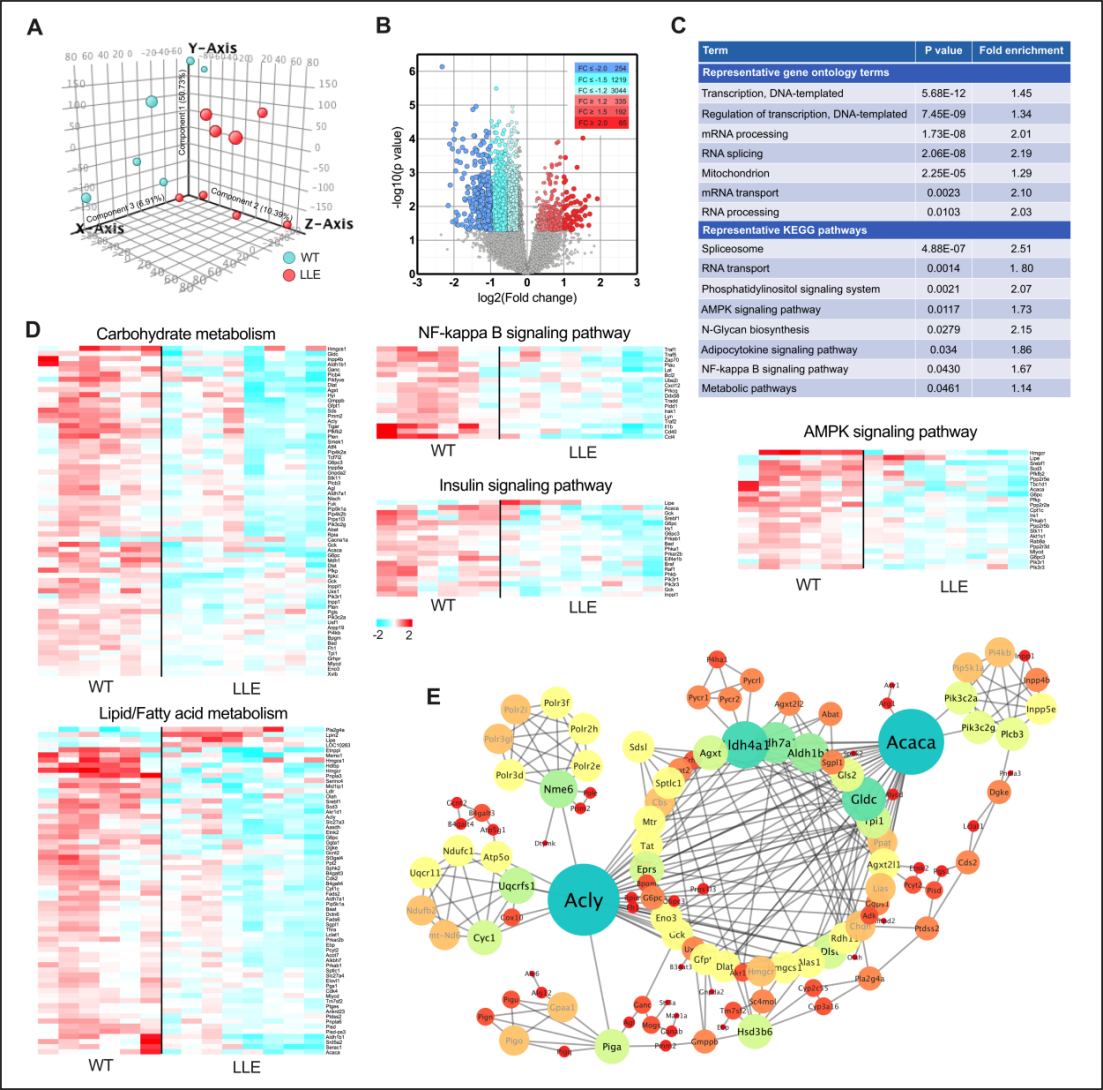
**Altered gene expression pattern in LLE mice.** (**A**) Principal component analysis (PCA) was performed on differentially expressed genes from the liver of 12-month old LLE mice. (n=6-8 for each group). (**B**) Volcano plot comparison of genes induced in LLE mice versus WT mice. (**C**) Gene ontology terms and KEGG pathway clustering of LLE mice. (**D**) Heatmap representation of genes involved in carbohydrate metabolism, lipid/fatty acid metabolism, NF-kappa B signaling, insulin signaling and AMPK signaling. (**E**) Protein-protein interaction network of differentially expressed genes of LLE mice.

Genome-wide microarray was performed to find the changes in gene expression in the liver of the LLE mice. Principal component analysis (PCA) showed a clear shift in the global gene expression profile caused by low-dose ethanol intake when compared with the WT mice (Figure 3A). Comparison between the LLE and the WT mice identified 3766 gene species that were differentially expressed in the liver (344 genes up-regulated and 3422 genes down-regulated. Fold change≥1.2, *P*<0.05; Figure 3B). Further ontological and pathway analyses revealed markedly distinct patterns between groups. Narrower sets of genes in categories ‘Transcription, DNA-templated’, ‘Regulation of transcription, DNA-templated’, ‘mRNA processing’, and ‘Spliceosome’ suggest that long-term low-dose ethanol may be directly involved in gene expression regulation (Figure 3C). Considering that alcohol is widely viewed as a carcinogen, of specific interest, we analyzed the connection between the differential genes and liver cancer. Unexpectedly, the upregulated genes of LLE mice showed no correlations with gene set of liver cancer (Supplementary Figure 2A), whereas the downregulated gene set of LLE mice are highly overlapped with the gene set overexpressed in live cancer (Supplementary Figure 2B), implying that moderate ethanol intake may have anti-cancer effects. Nevertheless, further experiments are needed to determine the relationship between low-dose alcohol intake and liver cancer.

Among differential gene enriched pathways are ‘AMPK signaling pathway’, ‘NF-kappa B signaling pathway’, ‘metabolic pathway’ etc. (Figure 3C–3D), which indicated that long-term low-dose ethanol intake is closely linked to energy metabolism, mitochondria function, and inflammation. Overall, the metabolic pathway is the most enriched pathway with 152 genes that are cohesively interconnected around two nodes, in spite of the large number (Figure 3E), both of which playing important roles in the metabolism of fatty acids [25]. The differential gene expression pattern of the LLE mice suggest that ethanol may be highly involved in the maintenance of lipid metabolism.

### Low-dose ethanol intake resists organ pathogenesis in mice on high-fat diet

**Figure 4:**
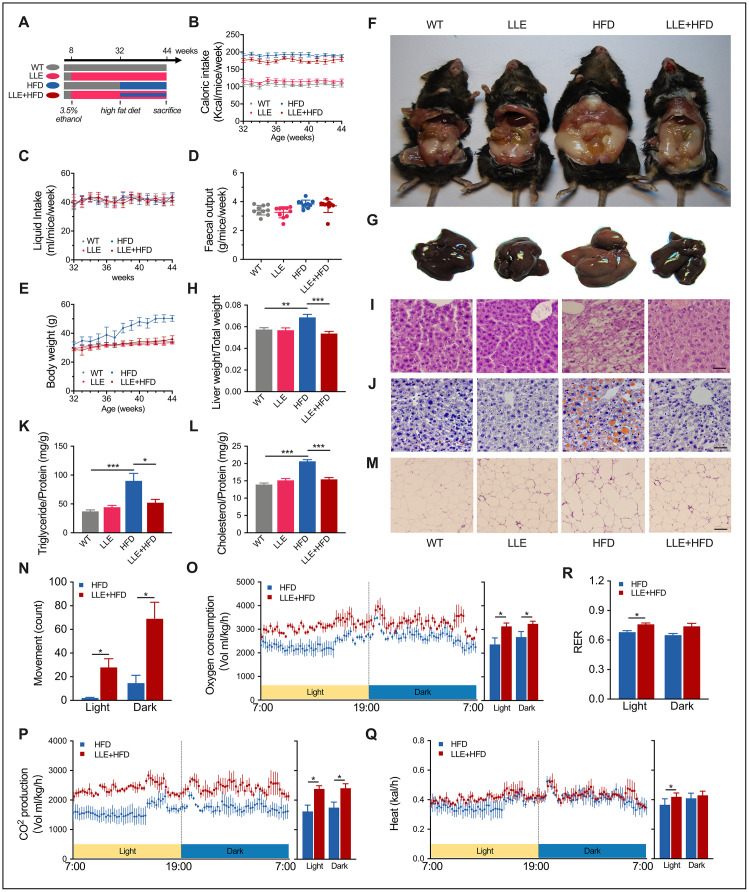
**Low-dose ethanol intake resisted HFD-induced organ pathology.** (**A**) Four group regimens. Mice fed with high-fat diet from week 32 to week 44. (**B**–**E**) Caloric intake, daily liquid intake, fecal output, and bodyweight of four groups (n=10 per group). (**F**) Representative anatomical images from four regimens after the 12-week treatment. HFD mice showed expanded visceral fat. (**G**) Representative images of mouse liver. HFD mice showed expanded fatty liver. (**H**) Liver weight ratio. (**I**–**J**) H&E staining and Oil Red O staining of liver after the 12-week treatment. Scale bar = 35μm. (**K**–**L**) Triglyceride and cholesterol level of liver (n=8 for each group). (**M**) H&E staining of adipose tissue. Scale bar = 70μm. (**N**–**R**) X-axis movement, Oxygen consumption rate, CO_2_ production rate, energy expenditure rate, and respiratory exchange ratio of HFD mice and LLE+HFD mice after the 12-week treatment (n=8 for each group). Data are presented as mean ± SEM. *, *P*< 0.05, **, *P*< 0.01, ***, *P*< 0.001.

It has been reported previously that long-term moderate alcohol consumption can significantly decrease fatty liver in humans [26], without acknowledging whether ethanol is the main substance responsible for the effect. The clustering of differential genes in LLE mice indicates the key role of ethanol on it. To test whether the ethanol can protect against injury that predisposes to obesity, a widely used rodent model of diet-induced obesity was adopted. We assigned the C57BL/6J mice into four experimental diet groups: WT group, LLE group, HFD group and HFD + LLE group. The HFD mice and LLE + HFD mice were fed with a 45% fat diet from week 32 to week 44 (Figure 4A). The nutritional profile was first determined by analyzing the caloric intake and body weight, the caloric intake per mouse was calculated as the weekly food intake multiplied by the dietary energetic value (Figure 4B). In this study, the liquid intake and fecal output of the four groups did not show any obvious difference (Figure 4C, 4D). Starting at similar baseline weights at 32 weeks, the body weight of HFD mice increased astonishingly after treated with the high-fat diet, but the LLE + HFD mice did not show much significant weight gain when compared with the WT group and LLE group (Figure 4E). By the end of the experiment, it was evident that a high-caloric diet greatly increased the body weight, liver size, and expanded viscera fat in the HFD group, while ethanol intake prevented these changes in the LLE+HFD group (Figure 4F–4H). Histological analysis on liver sections by haematoxylin-eosin staining and Oil Red O lipid staining showed signs of disorganized swollen hepatocytes and large lipid droplets-accumulated liver presented in the HFD mice. However, the liver of LLE + HFD mice was almost normal (Figure 4I, 4J). Unlike the mice fed with the standard diet, mice on the high-fat diet showed a distinct increase in their hepatic triglyceride and cholesterol levels, yet the LLE + HFD mice had significantly lower hepatic lipid and cholesterol levels in comparison (Figure 4K, 4L). In addition, the adipocytes expanded markedly in the HFD mice but not in the LLE + HFD mice, the WT mice, or the LLE mice (Figure 4M). Serum indicators such as triglycerides (TG), glucose (GLU), total cholesterol (T-CHO), high-density lipoprotein cholesterol (HDL-C), low-density lipoprotein cholesterol (LDL-C) and alanine aminotransferase (ALT) were elevated in the HFD mice. However, ethanol reversed these pathological changes in the LLE-HFD group (Table 1). Furthermore, the serum lactate dehydrogenase (LDH), a marker of common injuries and diseases that is released upon tissue damage, was elevated in the HFD mice but remained normal upon the supplementation of ethanol (Table 1).

Imbalance of energy metabolism caused by HFD is the primary reason for obesity. On the other hand, ethanol can promote physical activity and restore the balance of energy metabolism in the presence of HFD. Under the CLAMS monitor, we observed that the HFD mice prefer to stay quiescent but the LLE + HFD mice tend to be more active (Figure 4N). The higher oxygen consumption, carbon dioxide production, and heat production suggested the elevated metabolic rates and the exercise-lead high efficiency of energy utilization (Figure 4O–4R).

### Low-dose ethanol intake increases insulin sensitivity

**Figure 5:**
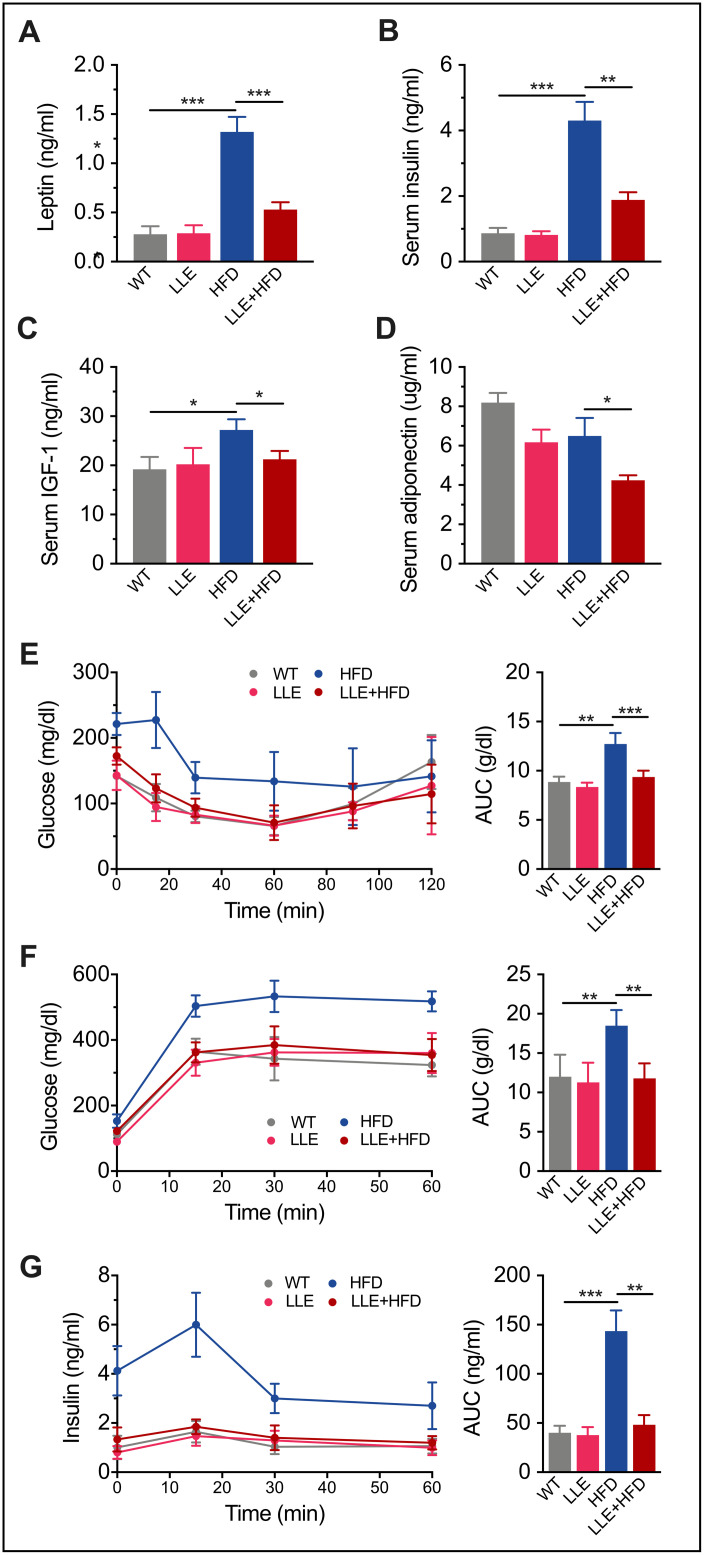
**Low-dose ethanol intake increased insulin sensitivity of HFD mice.** (**A**–**D**) Serum Leptin level, insulin level, IGF-1 level, and adiponectin level of four treatment regimens at week 44. (n=9 per group). (**E**) Plasma levels of glucose after intraperitoneal insulin injection (ITT) and area under curve (AUC) (n=9 per group). (**F**–**G**) Plasma levels of glucose and insulin measured after oral glucose tolerance test (OGTT) (n=7-10 per group). The tests were performed with 44-week-old mice under 12-weeks HFD/normal diet treatment. For oral glucose tolerance test (OGTT), mice were fasted overnight and gavaged with 2 g/kg of glucose. Insulin tolerance tests (ITT) were performed in nonfasted mice by IP injection of 1.5 IU/kg of insulin. Data are presented as mean ± SEM. *, *P*< 0.05, **, *P*< 0.01, ***, *P*< 0.001.

In humans, high-calorie diets cause numerous pathological conditions including obesity, cardiovascular disease, non-alcoholic fatty liver disease and diabetes. High levels of leptin in the HFD mice and increased plasma glucose, insulin and IGF-1 in the serum indicated leptin resistance and insulin resistance, suggesting the onset of diabetes (Table 1, Figure 5A–5C). In the HFD+LLE group, the levels of the same makers were significantly lower, comparable to those in the WT mice (Table 1, Figure 5A–5C), suggesting increased insulin sensitivity by ethanol. Moreover, the oral glucose tolerance test (OGTT) and insulin tolerance test (ITT) showed that the insulin sensitivity of the LLE + HFD mice was normal and considerably better than that of the HFD mice (Figure 5E–5G). These metabolic benefits of ethanol may be attributable to the activation of AMPK, a metabolic regulator that promotes insulin sensitivity and fatty acid oxidation. Surprisingly, the level of adiponectin was found to be the lowest in the LLE+HFD group (Figure 5D), against our initial expectations, considering its insulin-sensitizing properties. However, a recent report suggests that adiponectin deficiency may also rescue high fat diet-induced hepatic injury [27], leaving the role of adiponectin in this process to be further explored.

### Low-dose ethanol intake inhibits HFD-induced inflammation

**Figure 6:**
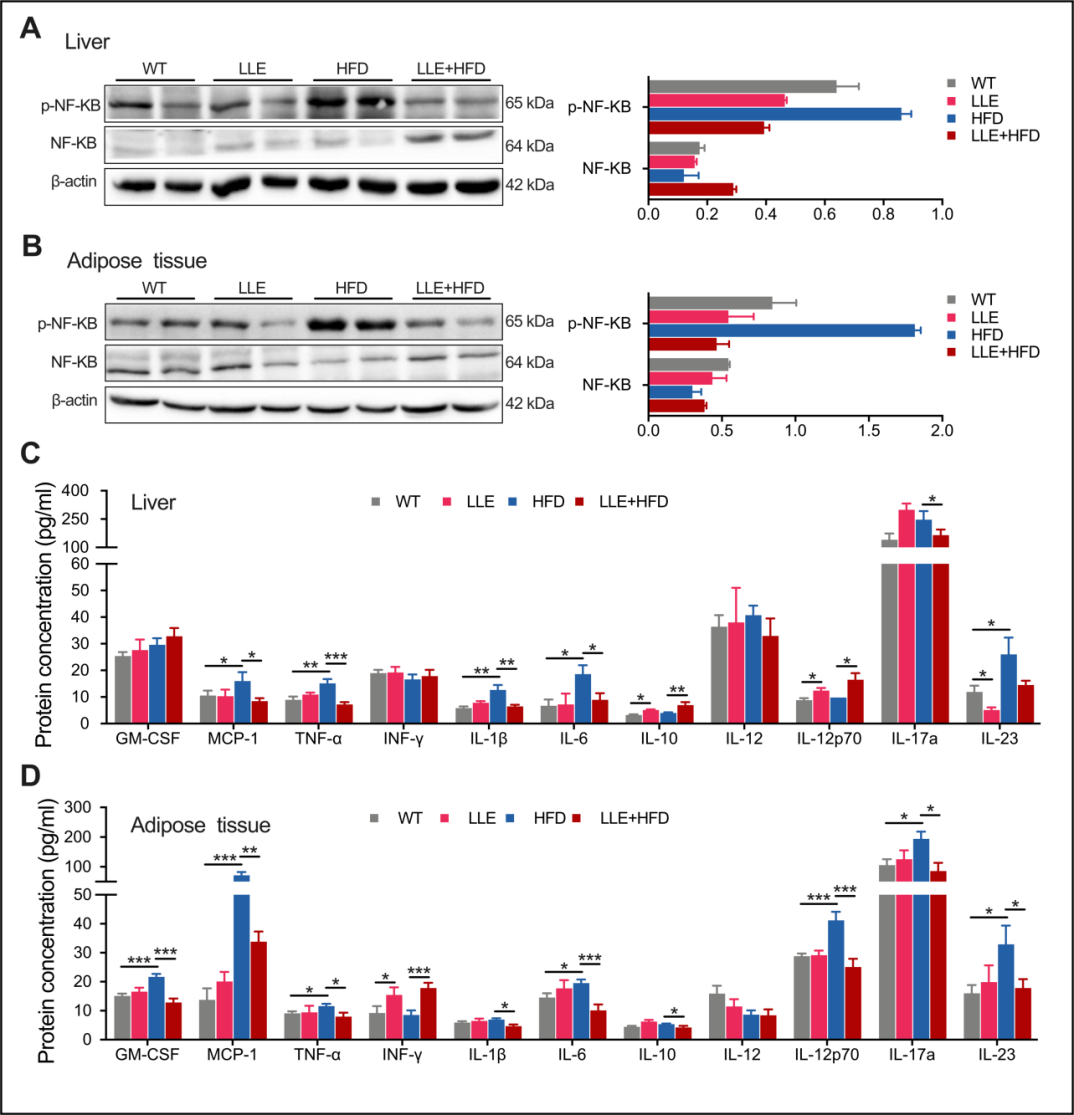
**Low-dose ethanol intake inhibited inflammation of HFD mice.** (**A**–**B**) Western blot analysis and relative quantification of NF-kB and p-NF-KB in liver and adipose tissue of 44-week-old mice (n=5-10 per group). (**C**–**D**) The Inflammation cytokine levels in liver and adipose tissue of 44-week-old mice. n=5-10 per group. Data are presented as mean ± SEM. *, *P*< 0.05, **, *P*< 0.01, ***, *P*< 0.001.

Chronic inflammation is the hub linking obesity with insulin resistance and type 2 diabetes. The development of chronic inflammation is one of the hallmarks of oxidative damage accumulation that is characterized by increased expression of proinflammatory cytokines. Increases in the level of phosphoactive form of NF-kB have been shown to contribute to pro-inflammatory signaling. Activation of NF-kB leads to the expression of further inflammatory mediators and results in a cycle of molecular events that lead to the fatty liver. In the LLE + HFD mice, a significant decline in p-NF-kB levels was observed in the liver and adipose tissue that was not seen in the HFD mice (Figure 6A, 6B). Monocyte chemoattractant protein 1 (MCP-1), the cytokine that considered interferes with the action of insulin and promotes insulin resistance and glucose intolerance [28], was also elevated in the liver and adipose tissue of the HFD mice but reduced in the LLE + HFD mice (Figure 6C, 6D). However, the pro-inflammatory cytokines such as TNF-a, IL-1β, IL-6 were decreased in the LLE + HFD mice but not in the HFD mice (Figure 6C, 6D). In summary, the ability for ethanol to reduce oxidative stress and inflammation suggested that it may be partly the reason why ethanol confers health benefits in mice.

## DISCUSSION

Previous studies on the protection of alcoholic beverages have been primarily focused on the polyphenols such as resveratrol, procyanidins and other substances like catechin and tannin [29–31]. Ironically, the most important common component of all alcoholic beverages, alcohol or ethanol, has received much less attention. Humans have had a long history of ethanol intake. The ‘drunken monkey hypothesis’ proposes that natural selection favored those primates with an attraction to ethanol because it was associated with proximate benefits [32, 33]. Even in modern times, scientists still observed the proactive behavior of wild chimpanzees taking ethanol [34]. In this study, we use ethanol, the common substance in all kinds of alcoholic beverages, as a single variable to explore its effects *in vivo*. Our data showed that the long-term 3.5% ethanol substitution for drinking water had beneficial effects in mice, the daily performance of ethanol-fed mice was enhanced, the athletic ability and healthspan of ethanol-fed mice drastically improved. Furthermore, the ethanol-fed mice showed the resistance to high-fat diet. When supplemented with 3.5% ethanol, the HFD mice showed reduced multiple organ pathogenicity, increased insulin sensitivity, and decreased NF-kB activation and inflammatory cytokines. These changes caused by ethanol are astonishing and impressive.

It has been well accepted that acute and chronic excessive alcohol exposure is conducive to tissue injury and that alcohol abuse is usually accompanied by a series of organ damages, including liver cirrhosis, cardiovascular disease, and cognition disorder, etc. [35–37]. However, one should be mindful that the injuries caused by the excessive use of alcohol are dose-dependent. In our study, the long term 3.5% ethanol-fed mice did not show the common negative effects of alcohol. At this dose, we did not observe any pathological structural changes in the liver, the heart, or the kidneys; neither did we detect any impairments of learning, memory, and cognition by the water maze. Previous epidemiology studies showed that moderate drinkers those who consumed less than 15.0 g of alcohol per day had better mean cognitive scores than nondrinkers in women [6]. On the other hand, a recent study also claimed that even moderate drinkers (14-21 units/week) had three times the odds of right-sided hippocampal atrophy and has no protective effect for light drinking (1-<7 units/week) [38]. However, the effects of moderate alcohol consumption on brain structure and cognition function need to be further explored.

One of the pathophysiological mechanisms induced by alcohol abuse has been identified as mitochondria dysfunction [39]. When the mitochondrial DNA damages induced by alcohol abuse are not adequately repaired, the mitochondrial function is impaired [40]. On the other hand, the mitochondrial volume was associated with high levels of physical activity [41]. The improved mitochondrial function of LLE mice may be due to their high level of daily physical activity and enhancement of athletic ability of LLE mice. In our experiments, we observed that the mitochondrial density in the liver and the skeletal muscles of the ethanol-fed group increased, and the morphology became stronger with more cristae, indicating improved mitochondrial function under the moderate ethanol feeding. AMPK induces mitochondrial biogenesis and has emerging roles in the regulation of both mitochondrial metabolism and dynamics [21, 22]. Phosphorylation activity of AMPK, necessary for mitochondrial biogenesis via SIRT1 and PGC1a [23, 24], was increased in the liver of the LLE mice. Considering the activation of AMPK by moderate ethanol intake, it seems reasonable to entertain the hypothesis that the rapid acetate metabolism following the ingestion of ethanol generates sufficient AMP to transiently activate AMPK, which in turn induces the synthesis of certain long-lived proteins that act to boost insulin sensitivity and possibly aid the efficiency of fat oxidation as well [42]. Furthermore, skeletal muscle contraction and exercise can stimulate the expression of AMPK [43, 44]. In our previous study, the peroxisome proliferator-activated receptors PPARα and PPARγ were found to be increased in the moderate alcohol-fed mice [17]. Both receptors are positively involved in mitochondrial biogenesis by promoting the transcription of upstream genes such as NRF-1, NRF-2, and Tfam [45, 46].

Obesity is commonly associated with insulin resistance, chronic systemic inflammation, and increased risk of cardiovascular disease [10]. The current consensus is that moderate alcohol consumption is associated with reduced risks of cardiovascular events, and can decrease the risks of type 2 diabetes [9, 12, 14, 15, 47, 48], partly owing to some of non-alcoholic components in the beverages. For instance, resveratrol in red wine has been shown to have anti-inflammatory properties, and can improve glucose tolerance and insulin sensitivity [49, 50]. However, the role of ethanol in the beneficial effects of moderate drinking is inadequately studied in comparison. As shown in our data, when supplemented with ethanol, HFD mice exhibited increased insulin sensitivity, lower level of inflammation, and decreased organ pathology, similar to the effects of resveratrol on HFD mice. These findings may explain why the beneficial effects of moderate alcohol drinking are not limited to red wine but also include most alcoholic beverages.

The health effects of alcohol intake are highly dependent on the amount of consumption, in addition to factors such as species (e.g., the alcohol metabolism rate of mice is faster than humans), gender, age, genotype (e.g., ALDH2 mutation), physical state [51]. A J-shaped dose-dependent relationship between alcohol consumption and effects has been proposed for human recently [52, 53]. In another study, Wood AM et al. analyzed 599912 current drinkers and recorded a positive and curvilinear association of all-cause mortality with the level of alcohol consumption, with the minimum mortality risk around 100 g per week [54]. For the aggregate of cardiovascular disease outcomes, a J-shaped association with the level of alcohol consumption was observed in the same study, with 100 g per week being the most beneficial dose [54]. By contrast, another study used 694 data sources of individual and population-level alcohol consumption, along with 592 prospective and retrospective studies on the risk of alcohol use, found that the risk of all-cause mortality, and of cancers specifically, rises with increasing levels of consumption, and asserted the level of consumption that minimizes health loss is zero [55]. The view on the beneficial effects of moderate drinking is inconclusive from these discussions. Furthermore, “moderate drinking” should be more clearly defined, and the long-term effects further explored with more rigorousness and scrutiny.

In conclusion, our findings showed that not only could long-term low-dose ethanol intake improve the physical performance and the healthspan in mice but also boost the defense mechanism against the high-fat diet. Extended evaluations are needed to assess the long-term impacts of moderate alcohol intake on organs or systems such as the brain, the muscular, and the cardiovascular system. Findings from the current study substantiate opinions on the protective effects of moderate alcohol intake.

## MATERIALS AND METHODS

### Mice

All animal experiments were approved by the Harbin Medical University Animal Care and Use Committee and were conducted according to the National Institutes of Health guidelines. C57BL/6J male mice were housed under a constant light/dark cycle in the SPF Mouse Barrier Unit. The wild-type (WT) group was allowed free access to food and water. For the LLE mice, ethanol (3.5% v/v) was supplied in the drinking water from week 8 to the end of life. In the HFD and HFD+LLE groups, the regular diet was replaced with a high fat diet containing 45 Kcal% fat (Research diets, D12451) for 12 weeks. Food intake and body weight were measured weekly for the duration of the study. Survival curves were plotted by using the Kaplan-Meier method, which included all animals available at each time point.

### Tissue processing and histology

For H&E staining, tissues were fixed in 4% formalin for 24 hours, dehydrated (Leica TP1020 automatic tissue hydroextractor, Leica, Germany) and embedded in paraffin (Leica EG1150C, Leica, Germany). 5-μm sections were collected and stained with haematoxylin/eosin. For Oil red-O staining, fresh mice liver was immediately frozen in Tissue-TEK OCT compound, 8-μm cryosections were collected and stained with Oil red O lipid stain. Electron microscopy analysis was performed at Harbin Medical University by the Electron Microscopy Group.

### Metabolic chamber analysis

The metabolic rate (VO_2_, VCO_2_ and heat production) and activity of mice were monitored by using an indirect open circuit calorimetry system (Comprehensive Lab Animal Monitoring System (CLAMS); Columbus Instruments, Columbus, OH, USA). Mice were acclimatized to monitoring cages for 6h before measurement and continuously measured for 24h, with measurements taken every 30s. Constant airflow (0.5 L/min) was drawn through the chamber and monitored by a mass-sensitive flow meter, the concentrations of oxygen and carbon dioxide were monitored at the inlet and outlet of the sealed chambers to calculate oxygen consumption. Light phase was from 7:00 to 19:00, dark phase was from 19:00 to 7:00.

### Treadmill and rotarod tests

The physical performance of mice was assessed by using treadmill and rotarod. For the treadmill test, mice were initially acclimated for 30 min for a warm-up and further familiarization with treadmill running. Mice were required to run at a relatively easy pace of 10 m/min for 30 min and then the speed of the treadmill was increased to 20 m/min, and the exercise duration and distance were recorded until exhaustion. For the rotarod test. Mice trained on the rotarod for 2 minutes at a speed of 10 rpm/min, which was increased by 1rpm/min every minute. The retaining duration was recorded until fall down.

### Blood chemistry

Mouse serum was obtained as the supernatant of the angular venous blood after 5 min of centrifugation at 5000 rpm. Samples were then analyzed by a System Chemix-180 automatic biochemistry analyzer (Sysmex Chemix-180, Sysmex, Japan).

### Quantitative real-time PCR

Total RNA was isolated using Trizol reagent (Invitrogen). cDNA was obtained using a high-capacity cDNA reverse transcription kit (Applied Bio-systems). Gene expression was quantified by an Applied Biosystems 7500 real-time PCR system using SYBR Green reagent (Roche) and mRNA-specific primers, and the β-actin mRNA levels were used for normalization.

### Oxygen consumption rate analysis

Oxygen consumption rate (OCR) was determined using a Seahorse XF24 Analyzer (Agilent Technologies). After sacrifice, liver and muscle were immediately rinsed with Krebs-Henseleit buffer and cut into 2mm diameter slices and transferred to individual wells of XF24 Islet Capture Microplate, covered with a customized nylon mesh by an islet capture screen insert tool. OCR was measured in response to oligomycin (1μM), FCCP (0.5 μM) and antimycin A (2 μM) plus rotenone (1μM). Basal respiration, maximal respiration, ATP production and spare respiratory capacity were calculated as previously described [56].

### Western blot

Tissues were homogenized and lysed by RIPA buffer. After centrifugation of the lysates at 12,000 g for 10 min at 4 degrees, the supernatants were collected. Equal amounts of protein were electrophoresed on SDS-polyacrylamide gel and transferred to a nitrocellulose membrane. The membrane was then preincubated with blocking solution (PBST containing 5% fat-free milk) for 2h before incubation with primary antibodies at 4 degrees overnight. The following primary antibodies were used: GADPH (1:4000; Abcam), β-actin (1:4000; Abcam), AMPK, (1:2000; Abcam). p-AMPK (1:2000; Abcam), ACC (1:2000; Abcam), p-ACC (1:2000; Abcam), SIRT1(1:2000; Abcam), PGC1a (1:2000; Abcam), NF-kB (1:2000; Abcam). After washing with PBST, the blot was incubated with secondary antibody (HRP-conjugated, 1:10,000 dilution) for 90 min and then washed and detected by an enhanced chemiluminescence detection kit (Haigene).

### Microarray

The Agilent SurePrint G3 Mouse GE V2.0 Microarray was used in this experiment. Total RNA was quantified by the NanoDrop ND-2000 (Thermo Scientific) and the RNA integrity was assessed using Agilent Bioanalyzer 2100 (Agilent Technologies). The sample labeling, microarray hybridization and washing were performed according to the manufacturer’s standard protocols. Briefly, total RNA was transcribed to double strand cDNA, then synthesized into cRNA and labeled with Cyanine-3-CTP. The labeled cRNAs were hybridized onto the microarray. After washing, the arrays were scanned by the Agilent Scanner G2505C (Agilent Technologies). Feature Extraction software (version10.7.1.1, Agilent Technologies) was used to analyze array images to get raw data. Genespring (version14.8, Agilent Technologies) was employed to finish the basic analysis with the raw data. Differentially expressed genes were then identified through fold change as well as *P* value calculated with t-test. The threshold set for up- and down-regulated genes was a fold change≥ 1.2 and a *P*-value≤ 0.05. Afterwards, GO analysis and KEGG analysis were performed to determine the roles of these differentially expressed genes. Protein interaction networks were analyzed by STRING (version 10.0) and arranged by Cytoscape (version 3.6.0).

### Triglyceride and cholesterol measurement

The hepatic triglyceride and total-cholesterol levels were determined using commercial kits (Applygen, Beijing, China). All experiments were performed according to the manufacturer’s instructions. The results were measured using a microplate reader (Infinite 2000 PRO, TECAN, Switzerland).

### Glucose and insulin tolerance test

Oral glucose tolerance test (OGTT) was performed after 8 weeks of regimens. Mice were fasted overnight and gavaged with 2 g/kg of glucose. Insulin tolerance tests (ITT) were performed in nonfasted mice by IP injection of 1.5 IU/kg of insulin. Blood glucose level was measured at 0, 15, 30, 60, 90, and 120 minutes. The insulin level was measured by an ELISA Kit (Crystal Chem Inc., Downers Grove, IL).

### Cytokine measurement

Cytokines in the supernatant were measured by LEGENDplex Mouse Inflammation Panel multi-analyte flow assay kit (BioLegend, San Diego, CA, USA), a bead-based immunoassay that quantifies multiple soluble analytes in biological samples simultaneously by flow cytometry (BD Biosciences). The assays were performed on 96-well plates, following the manufacturer's instructions.

### Statistical analysis

Microsoft Excel and GraphPad Prism (San Diego, CA) were used for statistical analysis. Data are presented as mean ± SEM. For longevity studies, Gehan–Breslow statistical test was used. Single comparisons between groups were examined with two-tailed Student’s t-test (normal distribution) or nonparametric Mann-Whitney test (non-normal distribution). Multiple comparisons were estimated by the analysis of variance (ANOVA) followed by Bonferroni’s multiple comparisons test. *P*<0.05 was considered to be statistically significant.

## Supplementary Material

Supplementary Methods

Supplementary Figures
