# Supplementary material for: Long-term low-dose ethanol intake improves healthspan and resists high-fat diet-induced obesity in mice
Source: Aging (Albany NY). 2020 Jul 8;12(13):13128–46. doi: 10.18632/aging.103401 (PMC7377878; doi:10.18632/aging.103401)
Supplement: Supplementary Methods [file aging-12-103401-s001..pdf]

SUPPLEMENTARY METHODS

Morris water maze test

The Morris water maze test was performed by previous reported method [1]. The pool was 100 cm in diameter and the water was rendered opaque by adding white dye. Water temperature was kept at 22-25 degrees. The platform (height 14.5 cm and diameter 4.5 cm) was submerged 1-1.5 cm below the surface of the water. The test consisted of four quadrants (from four different directions) to climb to the platform, and mice were trained for 5d during the acquisition phase (day 1-5). The position of the escape platform remained constant. In each quadrant the mouse swam until it found the platform, or after 90 s was guided to the platform; the mouse was then placed on the platform for 120s before being picked up. For the probe test, the platform was removed from the pool, and the mice were placed in the pool on the side and allowed to swim freely. At the end of the testing period, a probe trial (60s) was done. The swimming pattern of each mouse was monitored and recorded by a camera mounted above the center of the pool, and the escape latency, escape distance, and swimming speed were assessed with the SMART-LD program. A quiet environment, consistent lighting, constant water temperature, and fixed spatial frame were maintained throughout the experimental period. Statistical analysis was based on univariate and multivariate ANOVA and between-group comparisons by Tukey's Test.

REFERENCE

1. Vorhees CV, Williams MT. Morris water maze: procedures for assessing spatial and related forms of learning and memory. Nat Protoc. 2006;1:848–58. https://doi.org/10.1038/nprot.2006.116 PMID:17406317
