# Supplementary material for: Long-term low-dose ethanol intake improves healthspan and resists high-fat diet-induced obesity in mice
Source: Aging (Albany NY). 2020 Jul 8;12(13):13128–46. doi: 10.18632/aging.103401 (PMC7377878; doi:10.18632/aging.103401)
Supplement: Supplementary Figures [file aging-12-103401-s002..pdf]

SUPPLEMENTARY FIGURES

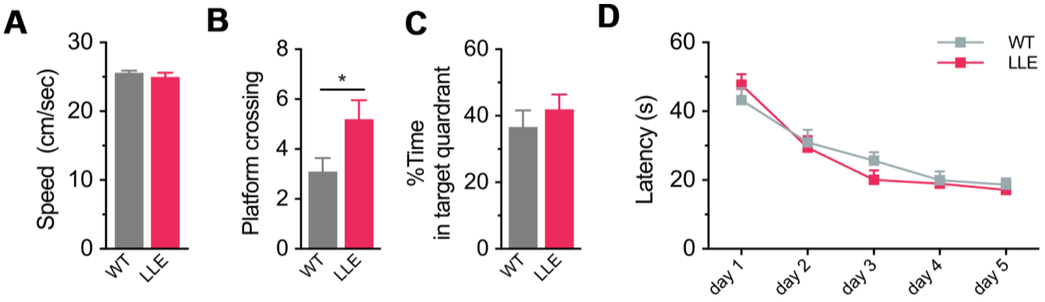

Supplementary Figure 1. Morris water maze test for LLE mice. (**A**) Swimming speeds averaged from all trials. (**B**) The number of crossings to platform on the probe trial. (**C**) Percent time spent in target quadrant. (**D**) Latency to reach hidden platform on training days. For each day of training, data were averaged across four daily trials. n=10. Data are presented as mean ± SEM. *, P< 0.05.

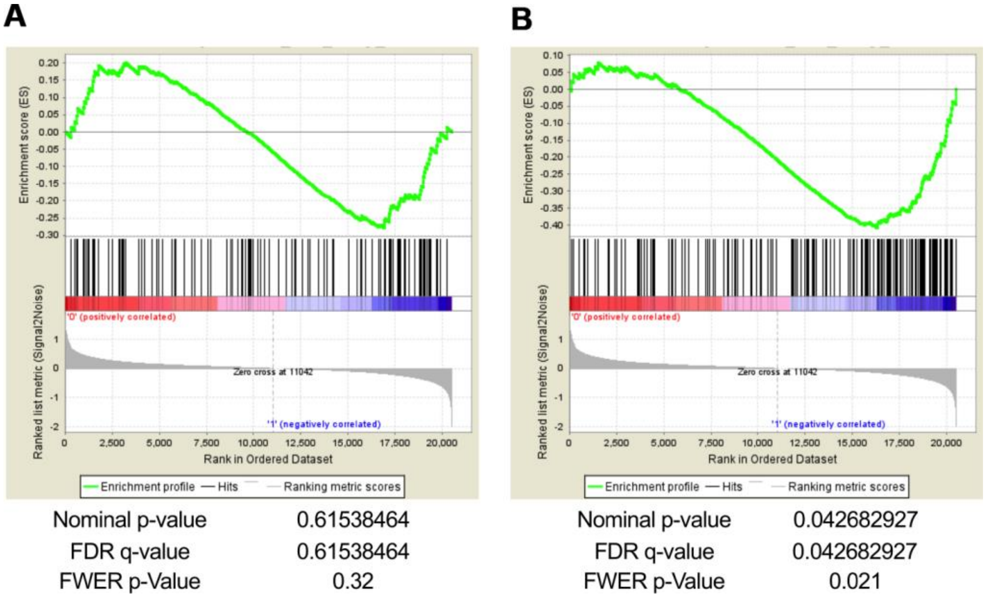

Supplementary Figure 2. Gene Set Enrichment Analysis for the differential genes in LLE mice. (**A**) The up-regulated gene set of LLE mice not enriched in metastatic hepatocellular carcinoma (HCC). (**B**) The down-regulated gene set of LLE mice enriched in metastatic hepatocellular carcinoma (HCC).
